# Technologies and Sensors for Artificial Muscles in Rehabilitation

**DOI:** 10.3390/s24237532

**Published:** 2024-11-26

**Authors:** Vina Basu, Li Cheng, Bin Zheng

**Affiliations:** 1 Department of Electrical Engineering, University of Alberta, Edmonton, AB T6G 2B7, Canada; lcheng5@ualberta.ca; 2 Department of Surgery, University of Alberta, Edmonton, AB T6G 2B7, Canada

**Keywords:** artificial muscles, muscle parameters and modelling, musculoskeletal functions, biosensors

## Abstract

Muscles are very important parts of the human body. When there is an injury to a muscle that causes long-term dysfunctionality, sensors and artificial muscles can be used to help alleviate problems. Muscles have complex structures; thus, ultrasound and other types of scans may be needed to determine their parameters and model their shapes. Additionally, the measurement of chemicals in muscles plays a significant role in analyzing their performance and potential diseases in humans. All the above-mentioned components are needed for understanding the structure and function of muscles. The areas studied in this review include artificial muscles and exoskeletons, determining muscle parameters and modelling, assessing musculoskeletal functions, chemicals in muscles, and various applications, including those of wearable sensors. In future studies, we would like to understand the link between the brain and muscles and develop technologies that can assist in augmenting the motor skills of individuals affected by various debilitating conditions.

## 1. Introduction

Muscles in the human body are a key component in controlling movement, as well as the functionality of various organs. We can better understand the complex structures and operation of muscles using sensors and technologies. When there are injuries or deficiencies in the muscles, specialized devices can be used to analyze the causes. Using these devices, we can take a closer look at damages in tissues to plan the best way to repair them. Many muscles cannot be fixed to function in their normal state before injuries. Thus, prosthetics or artificial muscles may be needed to assist in long-term rehabilitation.

Artificial muscles are not only meant to assist humans but can also be produced and tested on robots and exoskeletons. Creating artificial muscles, however, requires many detailed and precise steps. For example, a 3D ultrasound may be needed to create the model of a muscle and determine the muscle parameters. Following this, musculoskeletal functions need to be assessed so that a prosthetic is able to imitate the movement of a group of muscles.

Another aspect of the study of muscles is chemicals and their role in activities and diseases. The chemical energy is converted to mechanical energy when adenosine triphosphate (ATP), the source of energy for use and storage at the cellular level, is hydrolyzed. ATP is where energy is produced for our bodies to function. For example, the release of some chemicals in the muscles could be important in blood sugar levels. With high-tech sensors, one can now monitor glucose uptake in muscles and thereby assess the prospects of diabetes in patients. Electrical activities in muscles are also an important criterion to study. For example, electromyography (EMG) can measure the response of a muscle corresponding to a nerve’s stimulation of the muscle. EMG tests can be used to determine neuromuscular abnormalities, such as ALS and carpal tunnel syndrome.

The articles covered in this review were selected from a very large number in this field based on the following criteria: (i) they had some elements of technology in them, such as robotics and prosthetics; (ii) they utilized special sensors or fabricated customized sensors; (iii) they were related to human health or rehabilitation; or, (iv) they dealt with some applications of artificial muscles.

Considering the various aspects of muscles and related technologies discussed above, we have structured this review into the following five sections: In Section 2, the design and fabrication of artificial muscles are outlined. Their composition could include carbon nanotubes, sheath-run yarn, polymers, hydrogel, indium or fluorine-doped tin oxide, and stretchy fibres. Artificial muscles are now being used in new applications, such as monitoring civil structures and bridges and actuated catheters in surgery. Section 3 discusses some approaches to modelling and determining parameters for muscles. For example, using freehand 3D ultrasound and testing its ability to determine skeletal muscle volume in humans, which is a crucial indicator of muscle function, is outlined. Section 4 summarizes a few studies evaluating musculoskeletal functions. In Section 5, techniques for monitoring and measuring chemicals in muscles are described. Chemical activities, such as glucose uptake and calcium release, can play a vital role in determining physical conditioning and fatigue.

## 2. Artificial Muscles

Artificial muscles [1,2,3,4,5,6,7,8,9,10] inspired by real muscles have been researched for applications in robotics, prosthetics, and exoskeletons [1]. One type in particular, called carbon nanotube (CNT) yarn artificial muscle, has attracted special attention because of its special electrical and mechanical properties as an electrochemical artificial muscle. In [1], a large tensile stroke yarn-based artificial muscle is demonstrated by inducing increased capacitance. The CNT yarns following the biscrolling method produce greater tensile actuation, using more ions at the same voltage than pristine CNT coils. One limitation of this work is that no demonstration of the benefits is shown based on an actual person or application.

Even though CNT-based artificial muscles have good performance in terms of torsion and tensile strength, they are somewhat expensive. Considering this, an alternative technique was developed based on a sheath-run yarn [2]. It turns out that this technology has higher performance at a lower cost. For example, this type of muscle increases the maximum work capacity by a factor of 1.7 to 2.15 for tensile muscles. Sheath-run artificial muscles have 1.98 watts per gram of average contractile power, as well. A shortcoming of this work seems to be that tests were conducted using a prosthetic lower leg and not a real leg.

An overview of ionic polymer metal composites (IPMCs) for biomimetic sensors, actuators, and artificial muscles is presented in [3]. When an electric field is introduced, these IPM composites can undergo significant bending and flapping displacement. Thus, these sensors work like motion actuators that can be bent quasi-statically or dynamically. A voltage can be measured across the thickness of an IPMC strip. This voltage can be correlated to the applied load and calibrated for a sensor of standard size. Preliminary experiments show that there is a linear relationship between the imposed displacement and the output voltage. Several vibrational and resonance features in sensors and actuators can be designed using IPMCs. When the frequency is varied, large deformations of the displacements occur. Results have shown that the actuators exhibit good force-to-weight characteristics at low voltages. There seems to be great potential for this technology to be used for swimming, flying, and slithering robots, as well as devices for assisting in heart and circulation devices. However, building wing-flapping devices using this technology seems more of a concept.

Polymer and hydrogel hybrids have also been used for artificial muscles [4]. Hydrogel microfibers coated with conducting polymers can function as sensors for the concentration of an electrolyte. This is because the electrical energy consumed varies logarithmically with respect to the concentration of an electrolyte. The hybrid microfibers were fabricated using a chitosan solution and polymerized with pyrrole. The stability and the swelling/shrinking ability seem to be supported by the chitosan solution. This is expected to contribute toward biocompatibility in future biomedical applications. Even though new materials were developed and tested in this work, there is no study of how they can be placed on or utilized by human subjects.

Artificial muscles have also been used in other applications, such as the analysis of civil structures and bridges [5]. Researchers have determined that all modal parameters are not adequately excited by ambient forces; thus, artificial actuators need to be applied. One of the problems with large constructions is that they need big and heavy devices. As well, a large number of highly sensitive sensors require significant time for processing and analysis. Instead, this paper proposes an alternative using lightweight pneumatic artificial muscles combined with a scanning laser Doppler vibrometer to analyze structures. The new approach lowers the time needed for setup while supporting sine testing and the analysis of broadband signals. Experiments conducted to verify the approach seem to be in a laboratory setting and not actual bridges.

Emerging technologies that are semi-transparent and stretchable could be considered for artificial muscles and skin. Chemicals like indium tin oxide and fluorine-doped tin oxide have been used to create transparent conducting films. However, these materials do not have high conductivity when they undergo stretching and bending deformations. Thus, researchers have developed new conductive materials with wrinkled surface patterns with metal nanowires and carbon-based nanomaterials [6]. One of the promising nanomaterials is graphene. It has low sheet resistance and high transmittance and strength and can be stretched, which makes it a promising alternative for next-generation electronics. This specific paper discusses the fabrication of the new stretchable material but does not provide details on how the material can be used to construct and deploy artificial muscles.

A new type of electrochemical actuation of artificial muscles was conceived in [7]. This was achieved by electroactuating bipolar hydrogel/polyaniline microfibers by wet spinning a chitosan solution, followed by chemical polymerization. In response to the pH, the new material had enhanced chemical and electrochemical actuation. The response time and strain ratio depend on the pH of the electrolyte. The authors expect that the optimization of the fabrication conditions will result in better strains of fibres. They also anticipate that these fibres will be used to produce artificial muscles in the future. However, specifics on how the new fibres will be used to create artificial muscles are not outlined.

Artificial muscle technology has also found use in actuated catheters used in surgery. Catheters play an important role in minimally invasive surgery. Researchers are trying to produce controllable catheter tips that can be actively bent in various directions inside a patient’s body during surgery [8]. These types of tips can be navigated more effectively during surgery compared to those that are moved purely based on the force feedback sensed by surgeons on their hands during surgery. As the fabrication process and control mechanisms improve, these types of active catheters will enhance steering imaging probes and guidewires to reduce surgical times for cardiac, cerebral, and intravascular procedures. This work could be strengthened with some demonstration of the operation of the active catheter in a more realistic scenario, such as using an animal cadaver.

Some of the materials used to fabricate components of artificial muscles include stretchy fibres. In [9], the researchers discuss producing superelastic conducting fibres that can be used for electronics and sensors, as well as muscles. These highly stretchable sheath core conducting fibres were fabricated by wrapping carbon nanotube sheets on stretched rubber fibre cores. The fibres show noticeable sheath buckling that permits a resistance change of less than 5% for a stretch of 1000%. The authors demonstrated strain sensors that could generate an 860% capacitance change. Electrically powered torsional muscles were produced in the process. Another type of material used for artificial muscles included knitted CNT with spandex core elastomeric yarns [10]. These textiles can be tuned to different electrical conductivities. The resistance can change linearly depending on the tensile strength. The process also generates significant tensile contractions and has a maximum specific power output of 1.28 kW/Kg, far exceeding the specifications of the skeletal muscles of mammals. These properties make the material conducive to applications in smart clothing.

Based on the studies mentioned above, we could define artificial muscles as “A replica of biological muscles that can perform flexible actions like expansion, contraction, rotation, bending, stretching etc., with high power and strength relative to weight compared to natural muscles, conventional motors or pneumatic actuators”. Artificial muscles are an emerging technology and have great potential in medicine/rehabilitation, robotics, and various industrial applications. The movement of artificial muscles can be controlled by an external stimulus, such as the temperature, voltage/current, pressure, or chemical properties of the surrounding environment. It is conceivable that artificial muscles could be controlled by magnetic fields.

Table 1 summarizes some of the materials and methods scientists have used to create artificial muscles. 

Increasingly, polymers are being used in aerospace because of their low density and ease of adjusting their shape. One application of this is in high-performance structures and components for aircraft [11]. Soft, flexible actuators called pneumatic artificial muscles (PAM) can produce human-like actuation [12]. These make them ideal for medical applications and robotic platforms. Researchers have been exploring how to produce lightweight, flexible, and compact PAMS, including ones with high power-to-weight ratios. In short, biological muscles can be considered as an integration of sensors, actuators, and transmission elements, while artificial muscles are more like intelligent materials that respond to and deform with external stimuli [13].

An analysis of actuator technologies, along with their promising properties, were compared, outlining their developments and challenges. In this study, inertial sensors were used to evaluate the correctness of home-based rehabilitation exercises [14]. Tests on 17 physiotherapy patients helped assess the quality of rehabilitation exercise. The system was able to detect issues with exercise [15]. On the other hand, an experiment studied the effectiveness of three control systems, namely, proportional–integrative–derivative (PID), fuzzy logic controller (FLC), and sliding mode controller (SMC), in executing precise trajectory tracking using an exoskeleton. The FLC controller has the best global results [15].

Exoskeletons are robots that interact with humans and provide structural support. They are being used increasingly for rehabilitation, daily living assistance, performing augmentation, and as haptic devices. There has been considerable growth in research on exoskeletons, with sensors and actuation technologies being the fundamental themes [16]. Rehabilitation researchers are now entering a new era in which orthotic and prosthetic devices are becoming natural extensions of the human anatomy. Technologies for seamlessly merging the human and machine bodies are rapidly advancing, along with control systems that support related engineering mechanisms. The article in [17] discusses a state-of-the-art device for the rehabilitation of the leg. It is a powered ankle–foot orthosis for stroke, cerebral palsy, and multiple sclerosis patients. One major limitation is that the underlying technology, a force-controllable actuator, is heavy, noisy, and bulky. An alternative is electroactive, polymer-based artificial muscles [18]. These offer several advantages, such as joint impedance and motive force controllability, noise-free operation, and anthropomorphic device morphologies.

## 3. Determining Muscle Parameters and Modelling

A study using freehand 3D ultrasound has shown that this technology can be used to accurately determine skeletal muscle volume in humans [19]. Muscle volume is a crucial indicator of muscle function. A user study with eleven volunteers was conducted to compare the volumes for the quadriceps using MRI and ultrasound. The results of the study showed a mean difference of 0.53 cm^3^ and 95% agreement within ±2.24 cm^3^. In general, higher muscle volume is related to greater strength [20]. However, an increase in volume beyond a certain threshold seems to be ineffective.

The masseter muscle is one of four muscles responsible for mastication or chewing. In [21] the goal was to measure the volume, cross-sectional area, thickness, width, and length of the contracted masseter muscle using 3D ultrasonography. A magnetic positional sensor was used to integrate multiple 2D scans into a 3D volume of the muscle. The measures of various muscle parameters were consistent with those observed by previous investigators. One limitation of this study was the small number of subjects on which the study was conducted.

Muscle deformation was empirically quantified and modelled in [22]. Surface electromyography is often used for controlling biosignal-driven prostheses and exoskeletons. However, its noisy characteristics hinder acquiring distinguishable signal streams. Thus, researchers investigated the use of 2D B-mode ultrasound as another source of data relating to muscle activation. The process permitted the acquisition of multiple naturally varying signals that could be used for controlling high degree-of-freedom (DoF) assistive devices. A proof-of-concept study demonstrated that muscle deformations can be observed via ultrasound, and also showed the spatially varying characteristics of the signal. Improving assistive sensor design and validation are expected to materialize from this research.

The paraspinal muscle plays an important role in maintaining the dynamic stability of the spine. Segmentation is an important step in identifying different organs in the human body. In [23], the authors proposed a deformed U-Net to automatically segment paravertebral muscles in magnetic resonance images. They addressed the challenges of unclear boundaries of muscles, grey histogram distribution overlap, and varying intra- and inter-patient shapes. The architecture used a residual module and a feature pyramid attention (FPA) module to retain image details and extract salient features from high-level feature maps. Experiments using 120 cases from Shengjing Hospital, China Medical University, showed that the model attained higher predictive capability. Thus, the authors made a significant contribution toward the automatic measurement of paraspinal muscles. Another segmentation technique for abdominal muscles of polytrauma patients was based on deep learning (DL) U-Net [24]. This DL technique was trained to automatically segment muscles and adipose tissues from CT images. The training was performed using data from 3413 patients, and the testing was performed on 233 polytrauma patients. This work confirmed that U-Net architectures are capable of segmenting various types of muscles.

Why is estimating modelling and estimating muscle parameters important? The authors in [25] used electromyography (EMG) to measure parameters related to shoulder muscles. They showed that simple observations, like the sign of a muscle parameter signifying a positive or negative relationship between a parameter and the corresponding muscle force, could be used to predict muscle forces. In sports, for example, this type of analysis could help plan training programs for athletes. One can conceive that depending on the strengths of muscles in various parts of the body, a specific athlete could be suggested the optimal strategy to maximize the power of their shots. In general, the use of EMG in sports is quite broad, with many potential applications and limitations. The review in [26] throws some light on the vastness of this topic. Elliott [27] studied the biomechanics of stroke production in tennis. There is a general understanding that shoulder, chest, and upper body muscles play an important role in the power of shots in racquet sports. However, many others point out that the leg and back muscles are key to speed and agility and thereby improve the power and quality of shots [28]. The roles of various muscles have been analyzed for different sports by many authors. For example, in badminton, the coordination between hand and hip muscles in generating high-quality lobs is discussed in [29]. Thus, there are opportunities to build sophisticated models of muscles, estimate the parameters of muscles, and thereby predict the speed, agility, and power of athletes. These technologies can lead to more sophisticated and scientific training regimens for athletes that go well beyond subjective guidance provided by coaches in various sports. While some examples related to sports are discussed here, there are many other applications of muscle modelling and parameterization in rehabilitation and assistance of mobility for the disabled and elderly.

Another aspect that needs some discussion is devices that evaluate muscle functions. The development of lower limb exoskeletons for assistance in physical movement and rehabilitation was outlined in [30]. The article focused on the use of an actuation system, such as hydraulic actuators, electrical motors, series elastic actuators, and artificial pneumatic muscles. For these exoskeletons, control strategies are based on signals collected from the human body, human–robot interfaces, interaction forces between an exoskeleton and the person wearing it, and signals collected from the exoskeleton itself. The authors of [18] describe sensors and systems for rehabilitation and health monitoring. They highlight the rapid expansion of wearable equipment and sensing devices for physical activities. Three control systems, including proportional–integrative–derivative (PID), sliding mode control (SMC), and fuzzy logic controller (FLC), are composed based on effectiveness. This study aims to design, evaluate, and compare the task of executing precise trajectory tracking using an exoskeleton [15].

## 4. Assessing Musculoskeletal Functions

The musculoskeletal system [31,32] not only defines the structure of the human body and supports the means of movement but is also an endocrine system that is stimulated by exercise. It is also able to interact with bio-chemical signalling with different parts of the body. Interactions between bones and muscles are enhanced through exercise and help maintain good health. A lack of exercise leads to obesity and complications like diabetes. There are many studies on the positive effects of exercise on various aspects of the musculoskeletal system. In [33], the benefits of workplace exercises on work-related musculoskeletal disorders are studied. A later systematic review of this topic can be found in [34].

A study using electromyography (EMG) was described in [35] to evaluate musculoskeletal functions and the control of electrical prostheses. The approach has some limitations in distinguishing neighbouring muscles and collecting signals from deep muscles. Thus, the potential for using sonography to detect dimensional changes in muscles during certain actions was investigated. Dynamic ultrasounds from six young adults and three amputees were collected for forearm muscles. Sonomyography (SMB), or sonographically detected signals, could be associated with wrist angles. This demonstrated the potential for SMB in musculoskeletal control and assessment.

Evaluating skeletal muscle mitochondria dysfunction to understand the progression of pathologies or monitor muscle performance is an area of interest. In [36], a new medical device related to this topic was developed using time-domain near-infrared spectroscopy (TD NIRS). The device is a custom-printed 3D probe that contains optical elements capable of acquiring signals from muscles. The system was tested on solid phantoms, as well as healthy subjects. It was also tested for in vivo repeatability and acquisition on different muscles. In addition, a dynamic phantom was used to evaluate spatial and depth selectivity.

In recent years, specialized MRI and CT have been used to access musculoskeletal systems [37]. The goal has been to obtain higher-quality images faster and, at the same time, reduce exposure to radiation. To achieve this goal, deep learning algorithms are also being introduced to fill in details in under-sampled images. In addition, super-resolution is being applied to create 3D models from multiple 2D scans. The MRI and CT devices are now becoming more specialized in order to scan faster or reduce radiation exposure. For example, dual-energy CT, photon-counting detector CT, MR neurography, and energy-integrating detector CT are some of the technologies that are being utilized right now. Functional MRIs are being used to measure the persistence and recurrence of pain associated with musculoskeletal dysfunction [38]. Hybrid PET/MRI systems are also being used to access muscle functions [39]. In [40], dynamic MR spectroscopy (MRS) and MRI are used to study muscle functions, while body composition using CT and MRI is outlined in [41].

## 5. Chemicals in Muscles

The monitoring and measurement of chemicals in muscles can play a vital role in various types of analyses. Chemical release inside the muscle helps in metabolism, like creating energy supplies from glucose. For example, skeletal muscle tissues can control the glucose level in the body. Thus, impairments in these tissues can lead to diabetes. In [42], the authors developed a non-invasive glucose nanobiosensor to measure glucose uptake in skeletal muscle tissues. To enable the process, a culture medium that could keep muscle tissues alive for a long time was developed. A nanoporous gold biosensor was created to detect glucose uptake in this medium. The response of this sensor was linear depending on the concentration level of the glucose. Furthermore, it was observed that when the muscle tissue was electrically stimulated, the glucose absorption almost doubled in the first three hours. The authors expect their techniques to become a platform to test the efficacy of glucose biosensors and medical therapies in the future.

Calcium plays an important role in skeletal muscles. The depolarization of skeletal muscle fibres triggers the release of Ca^2+^ ions in the sarcoplasmic reticulum (SR) membrane [43]. Discrete localized release events known as “sparks” can occur from coordinated openings of ryanodine receptor (RyR) release channels or even single channels. These events can be activated by fibre depolarization or physiological cytosolic ligands in muscle fibres. Studying sparks gives us insight into the operation of groups of SR channels within a functioning fibre. The frequency and patterns of these sparks can be altered by varying the stimuli. Calcium release can be imaged using a laser-scanning confocal microscope [44]. Calcium release in skeletal muscles can trigger contractions at triads, which are special junctions in which SR channels are opened by voltage sensors in the transverse tubule. The concentration of intracellular calcium ions and their gradients were proportional to the calcium release. In cells stimulated with small depolarization, activities could be divided into small events that can be represented by 0.1 picoampere single-channel currents.

The level of myoplasmic Ca^2+^ in muscles and its potential effect on fatigue after severe exercise was studied in [45]. It was observed that elevated levels abolish depolarization-induced responses in skinned fibres from rats and toads in a concentrated and time-dependent manner. In addition, this dependence effect also occurs in intact muscle fibres, and uncoupling could have an important role in regulating muscle fibres by stopping excessive Ca^2+^ release. This study concludes that calcium-dependent uncoupling could be a very important step in understanding issues related to muscle function, exercise, and various diseases.

Calcium ion sparks in the large depolarization of skeletal tissues were studied in [46]. The work claimed to be the first recordings of these sparks using low repriming. The researchers inactivated all T1 voltage sensors and reprimed only a small number of them before each test depolarization. The low repriming helped in avoiding individual sparks being obscured by large transient ions. A related work [47] was the first to study spatially resolved Ca^2+^ release in adult mammalian muscles. Sparks were classified as multi-channel events. The study showed that developing mouse myotubes can produce these sparks.

The research in [48] revealed that there are two forms of calcium release in mammalian muscles. The first form is discrete, like the Ca^2+^ sparks released in cardiac muscles, while the second form is continuously induced by depolarization. A diffuse release that is similar happens for only a small segment of adult skeletal muscles. The two forms of release coexist in skeletal muscles, but they are segregated. Finally, two different mechanisms of quantized calcium release in skeletal muscles were identified in [49]. Skeletal muscles use voltage sensors in the transverse tubular membrane to control the release of calcium from the sarcoplasmic reticulum. By using voltage-clamped single fibres and confocal imaging, the authors showed that calcium release events originate in the triad and occur in skeletal muscles. For discrete calcium release events, the authors proposed a dual-control model.

## 6. Other Applications

Muscle movements have been used to control a cursor on a screen [50]. Infrared (IR) spectroscopy was used to monitor the neck muscles over time. Two IR photoplethysmography (PPG) sensors operating at a wavelength of 940 nm were used. The reflection of the IR light provided information on the type of movement, and two sensors were effectively used to predict the direction of movement. The approach was tested considering three scenarios, namely, low sensitivity, high sensitivity, and joystick mode. Experiments with four healthy adults demonstrated the feasibility of the method in terms of throughput, overshoot, and path efficiency.

Measuring muscle activities during exercise in a gym is an area considered in [51]. For this application, surface pressure changes between the skin and compression garments were measured using a wearable textile sensor system. The sensors covered the lower part of a person’s quadriceps. Considering various leg exercises and non-workout activities, the system attained a recognition rate of 81.7%. A tether-free prototype was also developed, which could be placed on areas such as the lower back, chest, and upper arm. The signal quality of the tether-free version was shown to be similar to arm-worn EMG.

Adding sensing to muscles is an even greater challenge than producing artificial muscles. In [52], the authors introduced new techniques to create a tactile sense. The oxidation of various types of films occurred through the formation of dark nuclei on reduced clear material. Digital pictures showed how these nuclei evolved. The process can be stabilized by switching off polarization. Changes to the composition of the materials affected the properties of the electro–chemo–mechanical conducting polymers. Artificial muscles in the form of sensors and actuators can demonstrate uniform and reverse movements, which can result in a tactile sense (or feeling of touch) with a potential gradient. A related work adding sensing and tactile components to artificial muscles was based on reactive materials [53]. Conducting polymer films can be oxidized and reduced reversibly, determining an electrochemical equilibrium. Chemical or physical parameters can modify this equilibrium. Acting as sensors, the performance of polypyrrole/dodecylbenzenesulfonic acid (DBSA) films changes depending on electrolyte concentration, temperature, or mechanical stress for constant currents. Devices defined by these properties act as sensors, such as artificial muscles that respond to ambient conditions. Finally, novel manufacturing processes for biomimetic sensors that can function as actuators and artificial muscles are described in [54].

A broad application area for technologies and measurements around muscles and musculoskeletal systems centres on sports. There is considerable research on this topic. We will outline a few in the following paragraphs.

In [55], a pneumatically powered orthosis for human ankle joints was built using a carbon fibre shell, a hinge joint, and two artificial pneumatic muscles. A low EMG amplitude was used to control the force of the muscles. Algorithms implemented by computer software enabled this process. Following a neurological injury, these artificial muscles can be useful in facilitating human locomotion and rehabilitation. The review in [56] focuses on the benefits of muscle strength on athletic performance. It emphasizes that greater muscle strength reduces injury risks while enhancing general sports skills and improving force-time characteristics. Isometric, dynamic, and reactive tests can be deployed by strength scientists and practitioners to monitor an individual athlete’s strength and performance. The authors suggest that there is no alternative to greater muscle strength when it comes to improving performance in general across a variety of sports skills. They also recommend that reducing sports injuries requires long-term muscle training.

The review article in [57] outlines the use of near-infrared spectroscopy (NIRS) in sports. NIRS can be used to evaluate the oxidative performance of skeletal muscles during sports activities, exercise, and training. However, NIRS currently has some “instrumental deficiencies”. Thus, the authors recommend that steps be taken to conduct longitudinal studies to determine the benefits of muscle oximetry in sports. Another review [58] also covers NIRS application in sports. Portability, optical imaging, and signal quantification have improved with advances in technology. During both full-body and localized exercises, oxidative metabolism in athletes can be measured using NIRS. Even chronic health conditions can be studied with this technology. Thus, NIRS can be used as a monitoring tool for various exercise and training interventions and measure oxidative capacity in muscles. One component that is still missing is a long-term longitudinal study to back up the suggested benefits of muscle oximetry. Another study [59] looked into the literature covering oxygen saturation in muscles during resistance training using wireless NIRS. The Downs and Black scale was used to evaluate the four studies that passed the evaluation criteria. SmO_2_ levels showed a decrease, both before and after the program, during muscle strength exercises.

Another topic that deserves some attention is wearable sensors. The assessment of recent developments in post-stroke rehabilitation with the help of wearable devices and machine learning algorithms is studied in [60]. The assessment systems are divided into activity regions, movement classification, and clinical assessment emulation. Common sensors, targeted body limbs, outcome measures, and study designs are reviewed throughout this paper. Monitoring older adults with chronic conditions in home and community settings is another focus of recent developments in wearable sensors and systems for rehabilitation [61]. Key enabling technologies in this approach include sensors, communication, and data analysis. This area is useful for the future of health and wellness, safety, home rehabilitation, treatment efficacy assessment, and the early detection of disorders. Stroke is a significant cause of long-term disabilities that affect many every year [62]. A wearable upper-body orthotic system is presented that is both cost-effective and empowering for patients/physiotherapists. The system consists of a lightweight, compliant soft orthotic device, an integrated cable actuation system, a limb position sensing system, and an electric actuator package. Successful actuation and online identification of misalignments were demonstrated through simulations and experiments.

Rehabilitation procedures are used to strengthen muscles when the ability to perform regular tasks is hampered by deformities in the hand [63]. Wearable technologies to accurately quantify rehabilitation can be divided into six categories, namely, using flex sensors, accelerometer-based, vision-based, applying the Hall effect, utilizing stretch sensors, and magnetic sensor-based. In this work, a wearable glove with enhanced features for better diagnosis and rehabilitation was proposed. In [64], AI processing and control systems in mobile robotic exoskeletons for upper-limb motor rehabilitation over 4 years found that 20% of the articles used adaptive algorithms, 40% used mixed-AI methods, and the remaining 40% used neural networks. The most observable trend seems to be the development of wearable robotic exoskeletons and multi-sensor data fusion.

A comprehensive overview of the application of wearable technologies during post-operative rehabilitation and athletic training is presented in [65]. The wearable hardware and sensors are used to detect and monitor physiological parameters in patients. They classify physiological parameters acquired from the human body using sensors placed on sensitive locations on the skin or worn on parts of different garments. In [66], wearable sensors and machine learning for disease-rehabilitation training programs are discussed. The study identifies the best sensors and algorithms for different scenarios and suggests some thoughts on future research directions. The discussions focus on types of wearable sensors, algorithms for machine learning, and approaches for rehabilitation. A combination of technologies and algorithms can optimize rehabilitation from diseases and offer more possibilities for home rehabilitation in the future.

## 7. Discussion

In this review, we looked at various types of technologies and studies related to muscles. For artificial muscles, carbon nanotube yarns are being researched for use in robotics, prosthetics, and exoskeletons. Ionic polymer metal composites (IPMCs) are being explored for biomimetic sensors, actuators, and artificial muscles. IPMCs can tolerate large bending and flapping displacement, making them suitable for motion actuators. Hydrogel and polymer hybrids are used for artificial muscles, with hydrogel microfibers coated with conducting polymers serving as electrolyte sensors. This work proposes using lightweight artificial muscles combined with a scanning laser Doppler vibrometer for structure analysis. Researchers are exploring semi-transparent and stretchable technologies for artificial muscles and skin, using chemicals like indium tin oxide and fluorine-doped tin oxide. Graphene is also being explored for electronics. Artificial muscle technology is being used to help surgeries by reducing surgical times and improving navigation. However, considering the relatively new use of artificial muscles, the fabrication can be quite costly, and these muscles can never completely replicate real biological muscles.

A study using freehand 3D ultrasound accurately determined skeletal muscle volume in humans, which is a very important indicator of muscle function. This research demonstrated the ability to observe muscle deformations using ultrasound. The authors have developed a deformed U-Net architecture to automatically segment paraspinal muscles in magnetic resonance images, thereby addressing challenges like muscle boundaries and varying shapes. Another U-Net technique was trained to segment the abdominal muscles of polytrauma patients, proving its ability to segment various types of muscles. Determining the skeletal muscle volume in our bodies helps the design and creation of artificial muscles by creating a close replication of an original biological muscle, although, as mentioned previously, these muscles cannot perfectly duplicate an original human muscle.

A study on musculoskeletal functions and electrical prosthesis control used electromyography (EMG). However, it has limitations in distinguishing neighbouring muscles and collecting signals from deep muscles. Some studies have investigated the potential of sonography to detect changes in muscles during specific actions. Dynamic ultrasound from young adults and athletes showed that sonographically detected signals could be associated with wrist angles. A new medical device, time-domain near-infrared spectroscopy (TD NIRS), was developed to evaluate skeletal muscle dysfunction and monitor muscle performance.

A non-invasive glucose nanobiosensor was developed to measure glucose uptake in skeletal muscle tissues. The control of glucose levels in tissues can potentially lead to diabetes. It was observed that when electrically stimulated, the glucose absorption in muscles increased. The researchers hope this technology will serve as a platform for future glucose biosensors and medical therapies. Calcium is crucial in skeletal muscles, as it triggers the release of Ca^2+^ ions in the reticulum membrane. These “sparks” can be triggered by fibre depolarization. Calcium release can trigger contractions, in which SR channels are opened by voltage sensors. One study explores the impact of Ca^2+^ on muscle fatigue after exercise. It found that elevated levels put an end to depolarization-induced responses in skinned fibres and muscle fibres. This suggests that calcium-dependent uncoupling could be crucial for understanding muscle function and disease. A study on calcium ion sparks in the large depolarization of skeletal tissues revealed two forms of calcium release in mammalian muscles: discrete and continuous.

Following the study of technologies related to muscles and musculoskeletal systems in general, it will be interesting to investigate the role of the brain and the “mind” in the control of muscles. In particular, we plan to look into the effect of brain injuries and mental health on human mobility. Going one step further, it will be beneficial to develop technologies that can assist in augmenting the motor skills of individuals affected by various debilitating conditions.

## Figures and Tables

**Table 1:** Methods and materials for artificial muscles.

Type of Artificial Muscle	Materials Used	Methods to Fabricate
Carbon nanotube	Materials with special electrical and mechanical properties composed of rolled up graphene sheets	The primary ways are chemical vapour deposition and laser ablation
Polymer and Hydrogel Hybrids	Conducting polymer coating, chitosan solution and polymerized pyrrole	Polymerization with the swelling and shrinking ability supported by chitosan solution
Electrochemical actuation	Hydrogel and polyaniline microfibers	Electroactuating bipolar hydrogel/polyaniline microfibers by wet spinning a chitosan solution, followed by chemical polymerization
Sheath core	Carbon nanotube sheets and rubber fibre	Wrapping carbon nanotube sheets on stretched rubber fibre cores

## Data Availability

Data are contained within the article.
